# Supplementary material for: Long first exons and epigenetic marks distinguish conserved pachytene piRNA clusters from other mammalian genes
Source: Nat Commun. 2021 Jan 4;12:73. doi: 10.1038/s41467-020-20345-3 (PMC7782496; doi:10.1038/s41467-020-20345-3)
Supplement: Supplementary file 1 — Supplementary Information [file 41467_2020_20345_MOESM1_ESM.pdf]

Supplementary Figures

Long First Exons and Epigenetic Marks Distinguish Conserved Pachytene piRNA Clusters from Other Mammalian Genes

Tianxiong Yu[1,2,†], Kaili Fan[1,2,†], Deniz M Özata[3], Gen Zhang[4], Yu Fu[2,5,#], William E. Theurkauf[4,*], Phillip D. Zamore[3,*] and Zhiping Weng[1,2,*]

[1]Department of Thoracic Surgery, Clinical Translational Research Center, Shanghai Pulmonary Hospital, The School of Life Sciences and Technology, Tongji University, Shanghai 200092, China

[2]Bioinformatics and Integrative Biology, University of Massachusetts Medical School, Worcester, MA 01605, USA

[3]RNA Therapeutics Institute, University of Massachusetts Medical School, Worcester, MA 01605, USA

[4]Program in Molecular Medicine, University of Massachusetts Medical School, Worcester, MA 01605, USA

[5]Bioinformatics Program, Boston University, 44 Cummington Mall, Boston, MA 02215, USA

[†]These authors contributed equally

*Correspondence: william.theurkauf@umassmed.edu (W.E.T), phillip.zamore@umassmed.edu (P.D.Z), zhiping.weng@umassmed.edu (Z.W.)

[#]**Present address:** Yu Fu, Oncology Drug Discovery Unit, Takeda Pharmaceuticals, Cambridge, MA 02139, USA

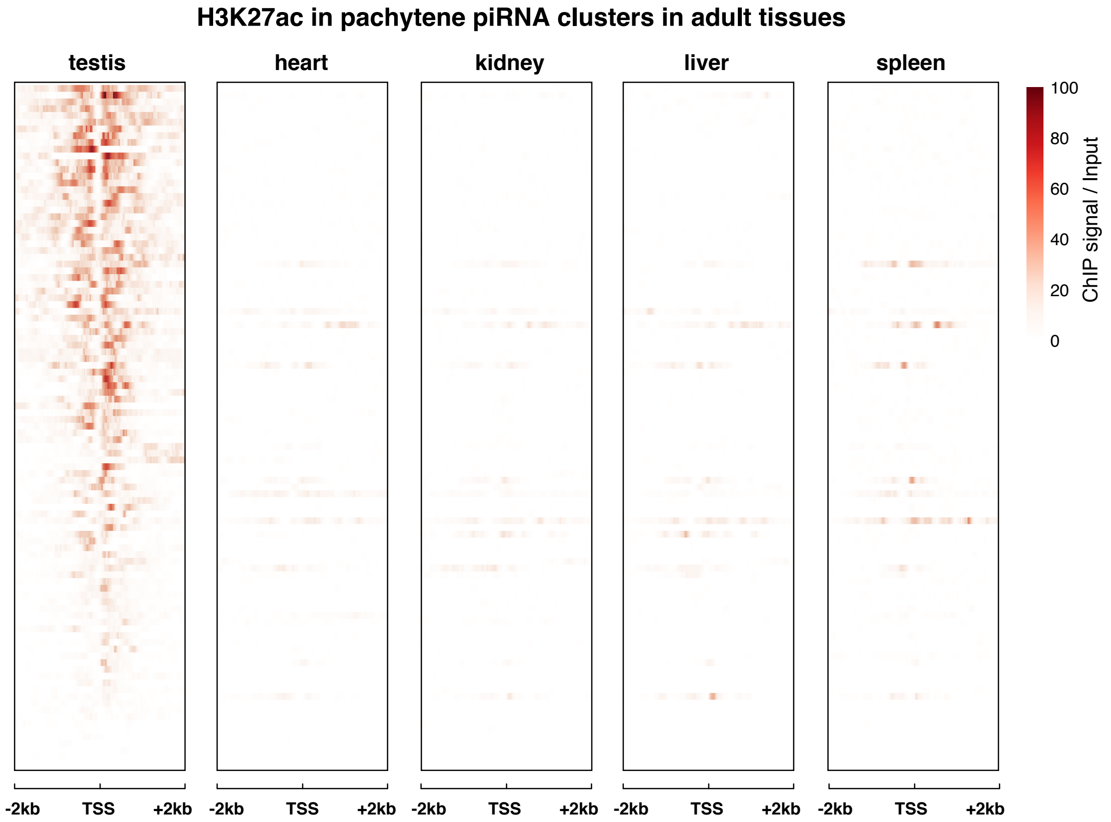

Supplementary Fig. 1 (related to Fig. 1). H3K27ac profiles at individual pachytene piRNA clusters. A heatmap shows the enrichment of the H3K27ac ChIP signal relative to input in adult testis, heart, kidney, liver, and spleen for 100 pachytene piRNA clusters in the −2 kb to +2 kb window flanking the TSS. Each pachytene piRNA cluster is represented by one row of the heatmap, in the same order across the tissues.

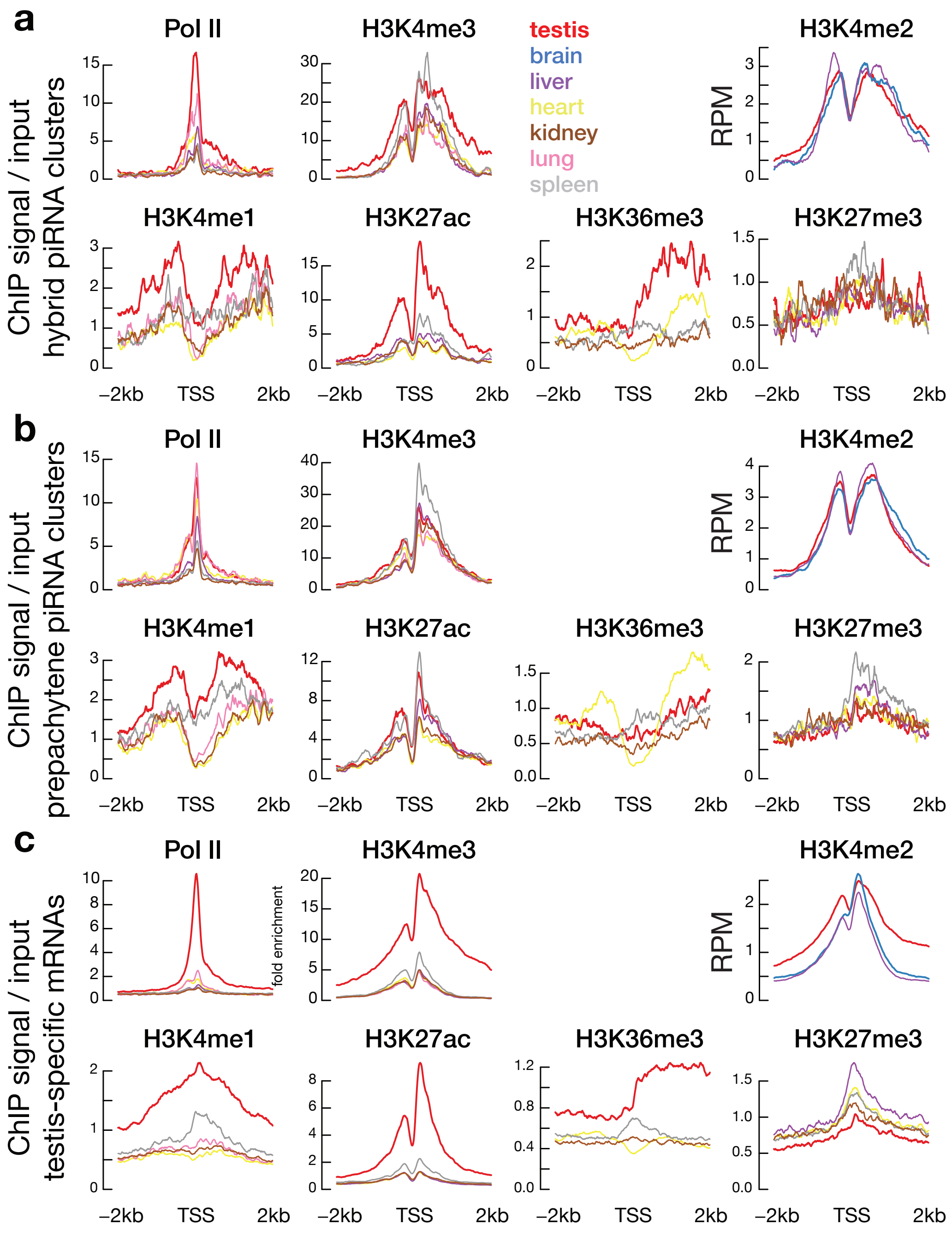

Supplementary Fig. 2 (related to Fig. 1). Epigenomic profiles of hybrid and pre-pachytene piRNA clusters and testis-specific protein-coding genes. These panels correspond to Fig.1b but are for **(a).** 30 hybrid piRNA clusters, **(b).** 84 pre-pachytene piRNA clusters, and **(c).** 1171 testis-specific protein-coding genes. Like pachytene piRNA clusters, the levels of RNA pol II, H3K4me3, H3K4me2, H3K4me1, H3K27ac, and H3K36me3 around the TSSs of testis-specific protein-coding genes

are significantly higher in testis than in somatic tissues (two-sided Wilcoxon signed-rank test p-value $< 2.2 \times 10^{-16}$), while the levels of H3K27me3 are significantly lower in testis than in somatic tissues (two-sided Wilcoxon signed-rank test p-values $< 2.2 \times 10^{-16}$).

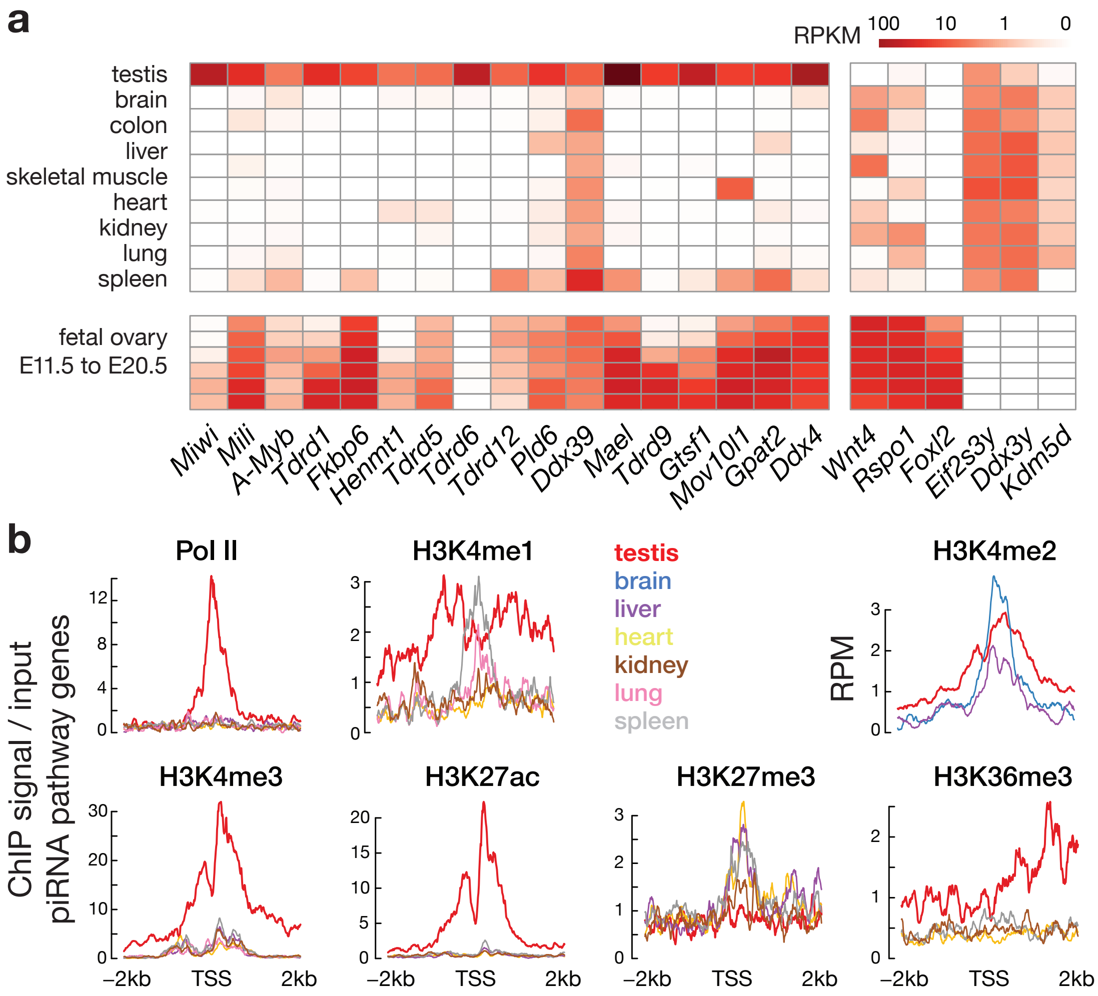

Supplementary Fig. 3 (related to Fig. 1). Protein-coding genes in the piRNA pathway are mostly testis specific. a. A heatmap shows the expression levels of 17 piRNA pathway genes in adult testis, eight somatic tissues, and fetal ovaries at embryonic days 11.5 to 20.5. *Ddx39* is ubiquitously expressed in somatic tissues, *Mov10l1* is also expressed in the heart, but other genes are not expressed in somatic tissues. Except for *Tdrd6*, the other 16 piRNA pathway genes are expressed in fetal ovaries. Three ovary-specific genes (*Wnt4*, *Rspo1* and *Foxl2*) and three Y-linked genes (*Eif2s3y*, *Ddx3y*, *Kdm5d*) are included as controls. **b.** These panels correspond to Fig. 1b but are for piRNA pathway genes. Like pachytene piRNA clusters, piRNA pathway genes exhibit higher levels of activating histone marks (H3K4me3, H3K4me2, H3K4me1, H3K27ac, and H3K36me3; two-sided Wilcoxon

signed-rank test *p*-values ≤ 4.8×10^{-4}), lower levels of the repressive histone mark H3K27me3 (two-sided Wilcoxon signed-rank test *p*-values ≤ 3.8×10^{-4}), and higher levels of RNA pol II binding in adult testis than in somatic tissues.

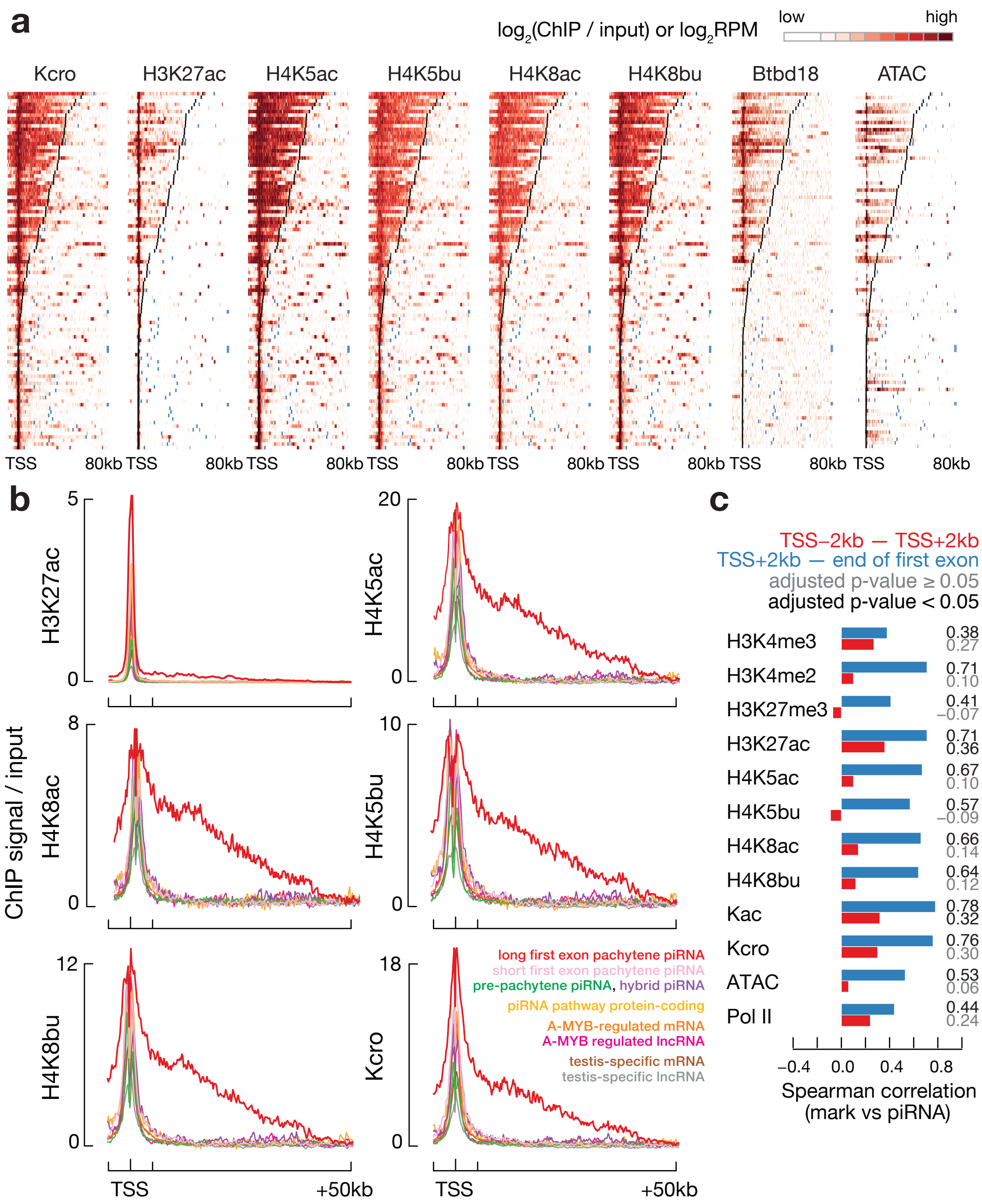

Supplementary Fig. 4 (related to Fig. 3). Long-first-exon or long intronless pachytene piRNA clusters bear high lysine acylation levels extending to the end of their first exons (or the end of the intronless gene). a-b. These panels corresponding to Fig. 3a-b but show Kcro, H3K27ac, H4K5ac, H4K5bu, H4K8ac, H4K8bu, BTBD18 binding, and chromatin accessibility (ATAC). **c.** A bar plot shows

the Spearman correlation coefficients between piRNA abundance and levels of epigenetic marks, including H3K4me3, H3K27me3, H3K4me2, H3K27ac, H4K5ac, H4K5bu, H4K8ac, H4K8bu, Kac, Kcro, chromatin accessibility (ATAC), and RNA pol II binding in pachytene spermatocytes for the 47 pachytene piRNA clusters with long-first-exons or are long intronless. The signal of epigenetic marks is calculated either at the first exon (2 kb downstream of TSS to the 3′-end of first exon of end of gene if intronless, in blue) or at promoter (TSS ± 2kb, in red). Significant correlations (Benjamin adjusted p-values < 0.05) are marked in black while non-significant correlations (Benjamin adjusted p-values ≥ 0.05) are marked in grey.

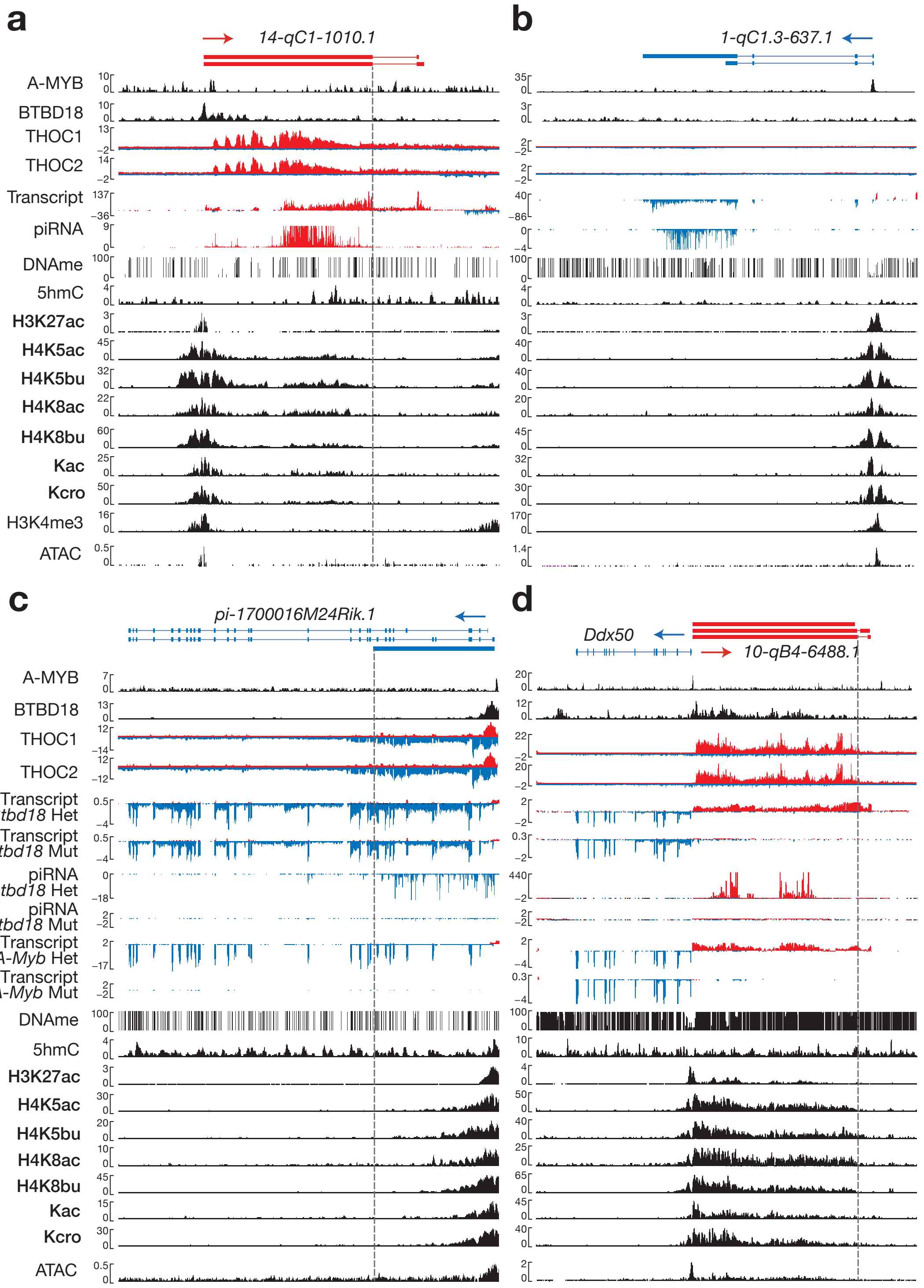

Supplementary Fig. 5 (related to Fig. 3). UCSC genome browser views for four additional examples of pachytene piRNA clusters. a. A long-first-exon pachytene piRNA-producing gene, *14-qC1-1010.1*. The end of the first exon is indicated with a vertical dashed line. A transposon insertion is immediately downstream of the TSS,

resulting in low mappability of sequencing reads there. **b.** A short-first-exon, high-CG pachytene piRNA-producing gene, *1-qC1.3-637.1*. *1-qC1.3-637.1* bears low DNA methylation and low 5hmC, as well as high lysine acylation but no BTBD18-binding at its promoter. **c.** A pachytene piRNA-producing gene, *pi-1700016M24Rik.1*, generates three isoforms with distinct fates. The same set of data as panel **c** are shown. The two short-first-exon isoforms are independent of BTBD18, while the other long and intronless isoform is dependent of BTBD18 for transcription and piRNA production. **d.** A pair of divergently transcribed genes: the long-first-exon pachytene piRNA-producing gene, *10-qB4-6488.1*, and the short-first-exon protein-coding gene, *Ddx50*. The transcription of *10-qB4-6488.1* is dependent on both BTBD18 and A-MYB, while *Ddx50* is independent of either. Transcription levels in *Btbd18* heterozygous pachytene spermatocytes, *Btbd18* mutant pachytene spermatocytes, *A-Myb* heterozygous 17.5 dpp testis and *A-Myb* mutant 17.5 dpp testis are shown. piRNA levels in *Btbd18* heterozygous pachytene spermatocytes and *Btbd18* mutant pachytene spermatocytes are also shown.

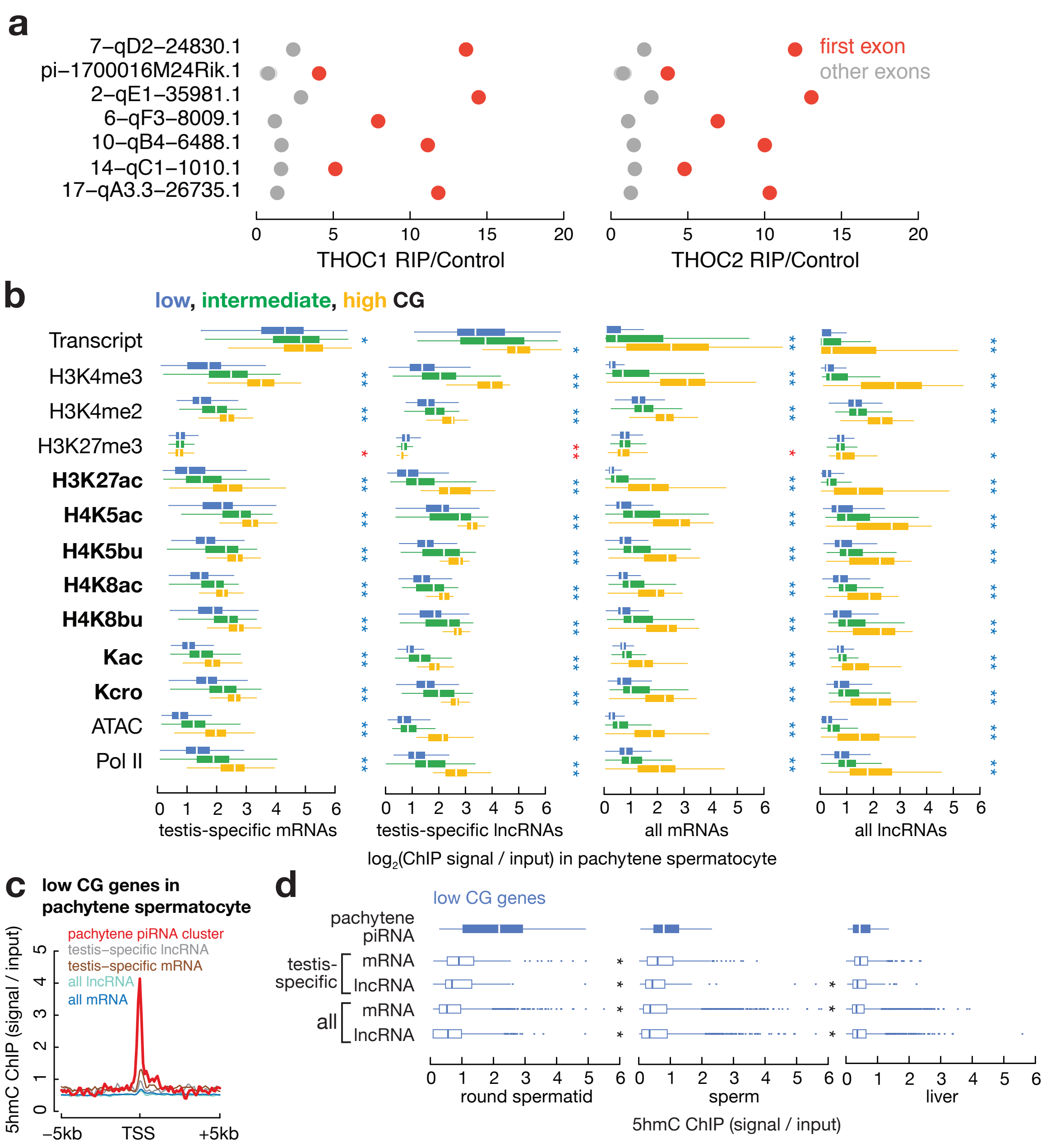

Supplementary Fig. 6 (related to Fig. 4 and 5). Pachytene piRNA clusters have high levels of histone acylation in pachytene spermatocytes, even for pachytene piRNA clusters with low-CG promoters. a. THOC1 and THOC2 RIP signals in the first exon and other exons for each of the seven spliced long-first-exon pachytene piRNA clusters. **b.** Histone acylation in pachytene spermatocytes, as in Fig. 5b but for genes including testis-specific mRNAs (n = 397, 369 and 405 for low-CG, intermediate-CG and high-CG promoters respectively), testis-specific lncRNAs

(n = 95, 40 and 29), all mRNAs (n = 6,177, 3,950 and 11,591), and all lncRNAs (n = 2,050, 888 and 525). For all boxplots in Supplementary Fig. 6, whiskers show 95% confidence intervals, boxes represent the first and third quartiles, and the vertical midline is the median. Asterisks indicate two-sided Wilcoxon rank-sum test *p*-values < 0.05. **c.** The average profiles of 5hmC signals around the transcription start site in pachytene spermatocytes among different types of genes with low-CG promoters. **d.** 5hmC levels for 49 pachytene piRNA clusters, 397 testis-specific mRNAs, 95 testis-specific lncRNAs, all 6,177 mRNAs, all 2,050 lncRNAs with low-CG promoters, as in Fig. 5c but in round spermatids, mature sperm, and adult liver.

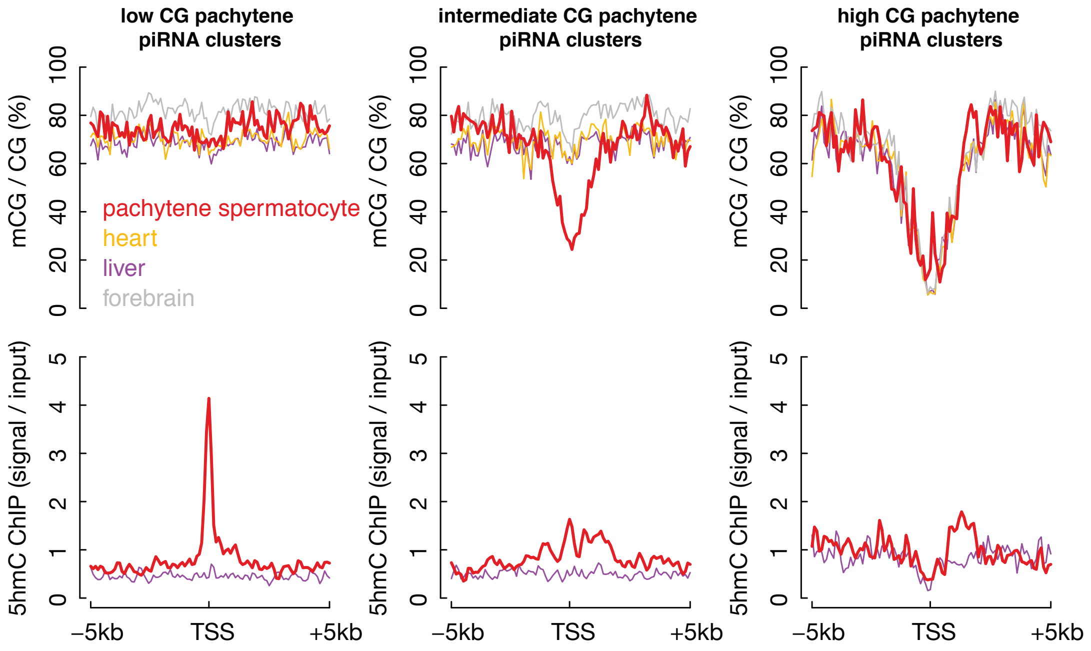

Supplementary Fig. 7 (related to Fig. 5). DNA methylation and 5hmC profiles of pachytene piRNA clusters. These panels show the average profiles of DNA methylation (top three) and 5hmC (bottom three) around the transcription start sites of low-CG, intermediate-CG and high-CG pachytene piRNA clusters in pachytene spermatocytes, heart, liver and forebrain.

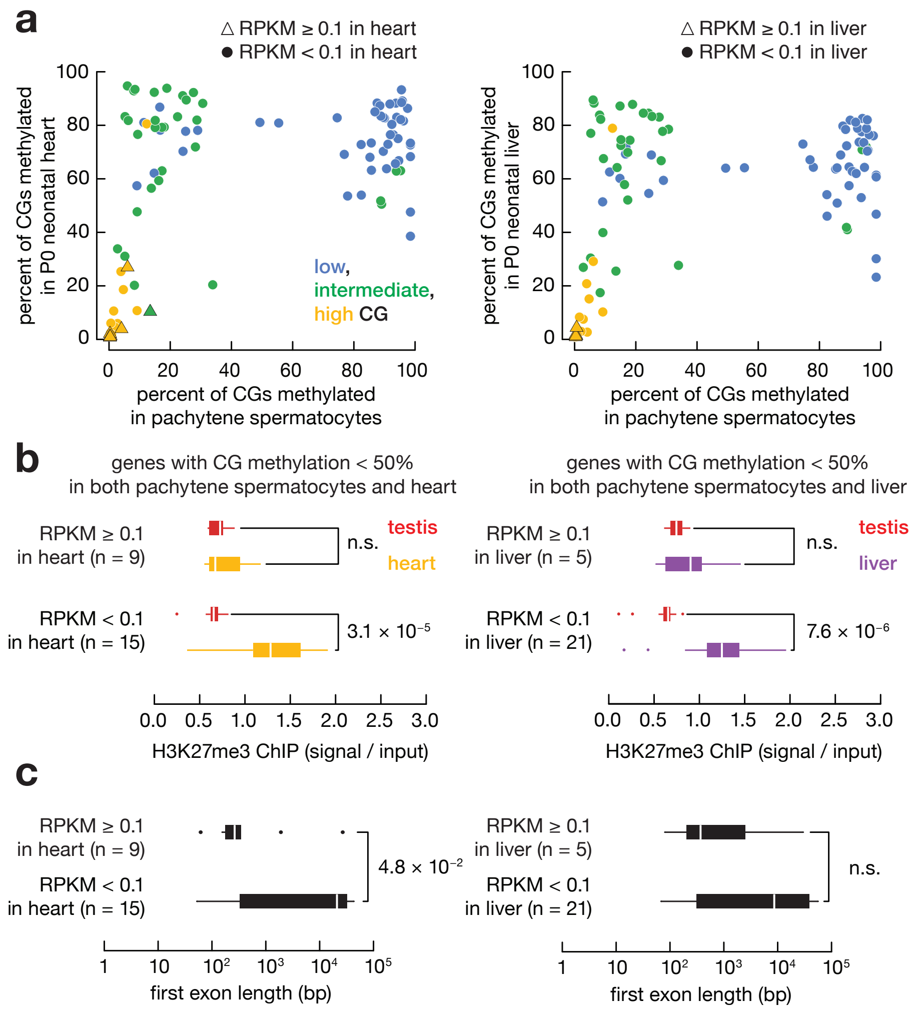

Supplementary Fig. 8 (related to Fig. 5). Multiple genic and epigenomic features correlate with the silencing of pachytene piRNA clusters in somatic tissues. a, **b**, and **c** correspond to Fig. 5e, f, g, respectively, but compare testis to heart (left panels) or liver (right panels). For all boxplots in Supplementary Fig. 8, whiskers show 95% confidence intervals, boxes represent the first and third quartiles, and the vertical midline is the median. Two-sided Wilcoxon signed-rank test **(b)** and Wilcoxon rank-sum test **(c)** are used to calculate *p*-values. n.s., not significant.

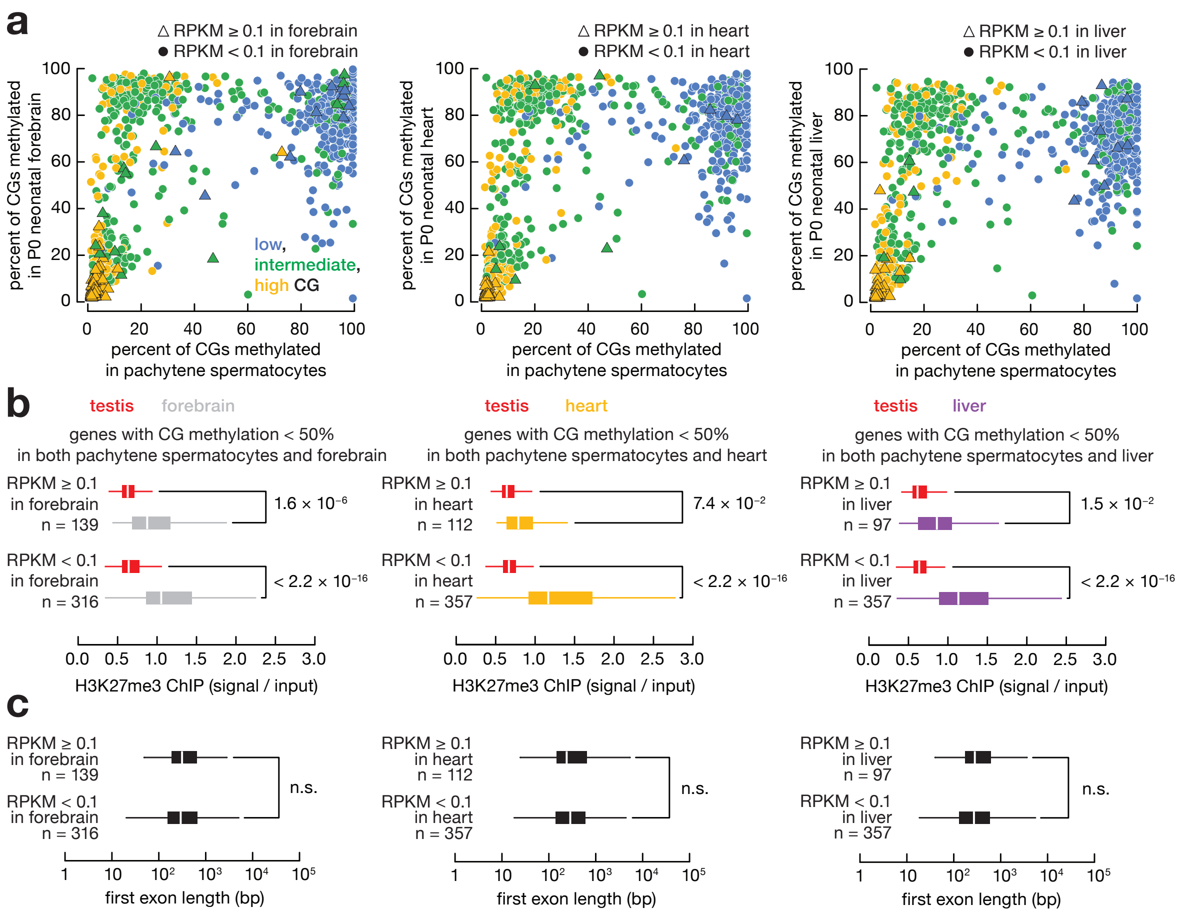

Supplementary Fig. 9 (related to Fig. 5). High DNA methylation and H3K27me3 levels correlate with the silencing of testis-specific protein-coding genes in somatic tissues. a, **b**, and **c** correspond to Fig. 5e, f, g, respectively, but for testis-specific protein-coding genes and comparing testis to forebrain (left panels), heart (middle panels) or liver (right panels). For all boxplots, whiskers show 95% confidence intervals, boxes represent the first and third quartiles, and the vertical midline is the median. Two-sided Wilcoxon signed-rank test **(b)** and Wilcoxon rank-sum test **(c)** are used to calculate *p*-values. n.s., not significant.

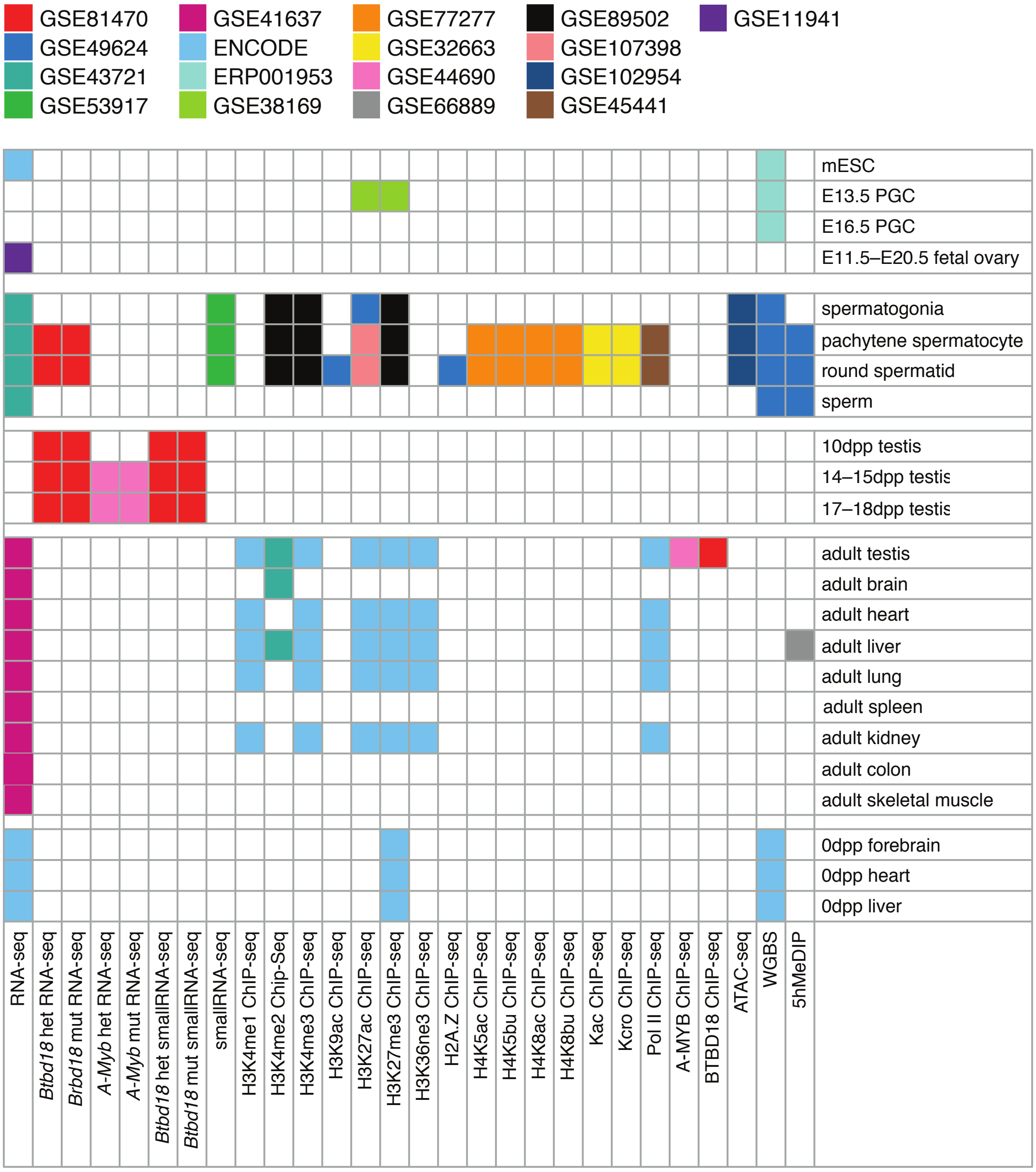

Supplementary Fig. 10 (related to Methods). Public data used in this paper. Colors depict different sources of the data and their accessions in the Gene Expression Omnibus (GEO) or ENCODE, while white indicates a lack of data.
