# Supplementary material for: Long first exons and epigenetic marks distinguish conserved pachytene piRNA clusters from other mammalian genes
Source: Nat Commun. 2021 Jan 4;12:73. doi: 10.1038/s41467-020-20345-3 (PMC7782496; doi:10.1038/s41467-020-20345-3)
Supplement: Supplementary file 3 — Reporting Summary [file 41467_2020_20345_MOESM3_ESM.pdf]

nature research

Corresponding author(s): William E. Theurkauf, Philip D. Zamore, Zhiping Weng

Last updated by author(s): Oct 24, 2020

# Reporting Summary

Nature Research wishes to improve the reproducibility of the work that we publish. This form provides structure for consistency and transparency in reporting. For further information on Nature Research policies, see Authors & Referees and the Editorial Policy Checklist.

## Statistics

For all statistical analyses, confirm that the following items are present in the figure legend, table legend, main text, or Methods section.

| n/a | Confirmed | |
|---|---|---|
| ☐ | ☒ | The exact sample size ($n$) for each experimental group/condition, given as a discrete number and unit of measurement |
| ☐ | ☒ | A statement on whether measurements were taken from distinct samples or whether the same sample was measured repeatedly |
| ☐ | ☒ | The statistical test(s) used AND whether they are one- or two-sided<br>*Only common tests should be described solely by name; describe more complex techniques in the Methods section.* |
| ☒ | ☐ | A description of all covariates tested |
| ☐ | ☒ | A description of any assumptions or corrections, such as tests of normality and adjustment for multiple comparisons |
| ☐ | ☒ | A full description of the statistical parameters including central tendency (e.g. means) or other basic estimates (e.g. regression coefficient) AND variation (e.g. standard deviation) or associated estimates of uncertainty (e.g. confidence intervals) |
| ☐ | ☒ | For null hypothesis testing, the test statistic (e.g. $F$, $t$, $r$) with confidence intervals, effect sizes, degrees of freedom and $P$ value noted<br>*Give P values as exact values whenever suitable.* |
| ☒ | ☐ | For Bayesian analysis, information on the choice of priors and Markov chain Monte Carlo settings |
| ☐ | ☒ | For hierarchical and complex designs, identification of the appropriate level for tests and full reporting of outcomes |
| ☐ | ☒ | Estimates of effect sizes (e.g. Cohen's $d$, Pearson's $r$), indicating how they were calculated |

*Our web collection on statistics for biologists contains articles on many of the points above.*

## Software and code

Policy information about availability of computer code

| | |
|---|---|
| Data collection | High-throughput sequencing:<br>Illumina NextSeq 500 System: https://www.illumina.com/ |
| Data analysis | Statistics and graph generation:<br>R (3.6.0): https://www.r-project.org/<br><br>High-throughput sequencing data analysis:<br>Bowtie2 (2.2.5): http://bowtie-bio.sourceforge.net/bowtie2/index.shtml<br>Bowtie (1.1.0): http://bowtie-bio.sourceforge.net/index.shtml<br>STAR (020201): https://github.com/alexdobin/STAR<br>HTSeq (0.9.1): https://htseq.readthedocs.io/en/master/<br>Trim Galore (0.4.1): https://github.com/FelixKrueger/TrimGalore<br>Bismark (0.15.0): https://www.bioinformatics.babraham.ac.uk/projects/bismark/<br>BEDTools (2.27.1): https://bedtools.readthedocs.io/en/latest/<br>MACS2 (2.1.1): https://github.com/taoliu/MACS<br>SAMtools (1.8): http://samtools.sourceforge.net/ |

For manuscripts utilizing custom algorithms or software that are central to the research but not yet described in published literature, software must be made available to editors/reviewers. We strongly encourage code deposition in a community repository (e.g. GitHub). See the Nature Research guidelines for submitting code & software for further information.

## Data

Policy information about availability of data

All manuscripts must include a data availability statement. This statement should provide the following information, where applicable:

- Accession codes, unique identifiers, or web links for publicly available datasets
- A list of figures that have associated raw data
- A description of any restrictions on data availability

The THOC2 RIP-seq data in adult mouse testis and the H3K27ac ChIP-seq data in adult rhesus testis have been deposited in the GEO with the accession GSE147724. All public data used in this study are downloaded from GEO and ENCODE and are shown in Supplementary Fig. 10 and Supplementary Data 3 with their accessions.

# Field-specific reporting

Please select the one below that is the best fit for your research. If you are not sure, read the appropriate sections before making your selection.

☒ Life sciences ☐ Behavioural & social sciences ☐ Ecological, evolutionary & environmental sciences

For a reference copy of the document with all sections, see nature.com/documents/nr-reporting-summary-flat.pdf

# Life sciences study design

All studies must disclose on these points even when the disclosure is negative.

**Sample size**

The investigators used at least 3 adult testis samples (3 for THOC2 RIP-seq, 5 for THOC1 RIP-seq, and 4 for IgG control RIP-seq) for the analysis of THOC1/THOC2 binding at pachytene piRNA genes, to achieve enough statistical significance

**Data exclusions**

No data are excluded.

**Replication**

We validated the reproducibility of our data using 5 replicates for THOC1 RIP-seq, 3 replicates for THOC2 RIP-seq, and 4 replicates for IgG Control RIP-seq. The Spearman and Pearson correlation co-efficiencies between replicates are > 0.99. Thus, we confirmed that all attempts of replications are successful.

**Randomization**

Animals used in this study were grouped randomly, involving no subjective animal allocation.

**Blinding**

The investigators are blinded in all the experiments other than sequencing experiments. For sequencing experiments, blinding is not relevant. Libraries from wild-type and mutant animals are sequenced in the same run when possible to minimize artificial differences created by separate sequencing runs, which requires prior knowledge of the sample identities.

# Reporting for specific materials, systems and methods

We require information from authors about some types of materials, experimental systems and methods used in many studies. Here, indicate whether each material, system or method listed is relevant to your study. If you are not sure if a list item applies to your research, read the appropriate section before selecting a response.

### Materials & experimental systems

| n/a | Involved in the study | |
|---|---|---|
| ☐ | ☒ | Antibodies |
| ☒ | ☐ | Eukaryotic cell lines |
| ☒ | ☐ | Palaeontology |
| ☐ | ☒ | Animals and other organisms |
| ☒ | ☐ | Human research participants |
| ☒ | ☐ | Clinical data |

### Methods

| n/a | Involved in the study | |
|---|---|---|
| ☐ | ☒ | ChIP-seq |
| ☒ | ☐ | Flow cytometry |
| ☒ | ☐ | MRI-based neuroimaging |

## Antibodies

**Antibodies used**

rabbit polyclonal anti-H3K27Ac antibody (Abcam, ab4729)
mouse anti-THOC1 (NB100-174 Novus)
mouse anti-IgG (12-371 EMD millipore)
Rabbit anti-THOC2 (Abcam, ab129485)
Rabbit anti-IgG (12-370 EMD millipore)

**Validation**

All antibody are either purchased from a validated commercial source or obtained from published source. We validated the anti-H3K27ac antibody specificity by checking whether the majority of peaks called from the H3K27ac ChIP-seq data are located at

the promoters of protein-coding genes.. We also validated anti-THOC1, anti-THOC2 and anti-IgG antibodies by checking the enrichment of RIP-seq signals in protein-coding transcripts.

# Animals and other organisms

Policy information about studies involving animals; ARRIVE guidelines recommended for reporting animal research

| | |
|---|---|
| Laboratory animals | C57BL/6J mice: JAX# 000664; wild-type control for all experiments; 2–4 month old males |
| Wild animals | No wild animals are used in this study. |
| Field-collected samples | No field-collected samples are used in this study. |
| Ethics oversight | The Umass Medical School (UMMS) Institutional Animal Care and Use Committe (IACUC) approved the animal procedures under protocol D16-00196 on 16 February 2017. |

Note that full information on the approval of the study protocol must also be provided in the manuscript.

# ChIP-seq

## Data deposition

☒ Confirm that both raw and final processed data have been deposited in a public database such as GEO.

☒ Confirm that you have deposited or provided access to graph files (e.g. BED files) for the called peaks.

| | |
|---|---|
| Data access links<br>*May remain private before publication.* | https://www.ncbi.nlm.nih.gov/geo/query/acc.cgi?acc=GSE147724 |
| Files in database submission | 1. Raw reads; 2. Signal file in bigWig format. |
| Genome browser session<br>(e.g. UCSC) | https://genome.ucsc.edu/s/yutianxiong/pachytene_project_screenshot |

## Methodology

| | |
|---|---|
| Replicates | 1 |
| Sequencing depth | We acquired 25.3 million paired-end 79nt reads for H3K27ac ChIP-seq experiment.<br>We acquired 22.4 million paired-end 79nt reads for input experiment. |
| Antibodies | rabbit polyclonal anti-H3K27Ac (Abcam, ab4729). |
| Peak calling parameters | No peak calling was performed |
| Data quality | We measured H3K27ac enrichment in the promoters of protein-coding genes, lncRNAs and piRNA genes in rhesus macaque. The enrichment is significantly higher than intergeneic regions, indicating high data quality. |
| Software | Bowtie2, MACS2 |
