# Supplementary material for: Long first exons and epigenetic marks distinguish conserved pachytene piRNA clusters from other mammalian genes
Source: Nat Commun. 2021 Jan 4;12:73. doi: 10.1038/s41467-020-20345-3 (PMC7782496; doi:10.1038/s41467-020-20345-3)
Supplement: Supplementary file 4 — Description of Additional Supplementary Files [file 41467_2020_20345_MOESM4_ESM.pdf]

Description of Additional Supplementary Files

File Name: Supplementary Data 1
Description: The expression level (RPKM) of protein-coding genes and piRNA clusters from all RIP-seq data used in this project. The matrix also contains change, p-value and FDR of THOC1 RIP over IgG RIP. Two-sided Wilcoxon signed-rank test was used to calculate p-value and Benjamini & Hochberg adjustment is applied for multiple comparison.

File Name: Supplementary Data 2
Description: The normalized signal matrix of all ChIP-seq, RNAseq, small RNA-seq, 5h-MeDIP, and WGBS data used in this project. The matrix contains 46,287 rows (21,774 protein-coding genes, 3,463 lncRNAs, 214 piRNA clusters, and other non-coding genes). The matrix also contains the information on genomic features including first-exon length, promoter O/E CG as well as the type of each gene (e.g., whether it is A-MYB–regulated or testis-specific).

File Name: Supplementary Data 3
Description: Mapping statistics and accessions for RNA-seq, ChIPseq and small RNA-seq data used in our analysis.

File Name: Supplementary Data 4
Description: Refined definitions of piRNA clusters for mouse, rhesus, marmoset, rat, cow, opossum, and platypus
